# Hemorrhagic transformation during inter-hospital transfer for thrombectomy: Incidence, associated factors, and relationship with outcome

**DOI:** 10.1093/esj/23969873251349713

**Published:** 2026-01-01

**Authors:** Pierre Seners, Adrien Ter Schiphorst, Anke Wouters, Nicole Yuen, Michael Mlynash, Caroline Arquizan, Jeremy J Heit, Denis Sablot, Anne Wacongne, Thibault Lalu, Vincent Costalat, Gregory W Albers, Maarten G Lansberg

**Affiliations:** Neurology Department, Hôpital Fondation A. de Rothschild, Paris, France; Université Paris Cité, Institute of Psychiatrie and Neurosciences of Paris (IPNP), INSERM U1266, Team Turc, Paris, France; Stanford Stroke Center, Palo Alto, CA, USA; Université Paris Cité, Institute of Psychiatrie and Neurosciences of Paris (IPNP), INSERM U1266, Team Turc, Paris, France; Stanford Stroke Center, Palo Alto, CA, USA; Neurology Department, CHU Montpellier, Montpellier, France; Stanford Stroke Center, Palo Alto, CA, USA; Division of Experimental Neurology, Department of Neurosciences, KU Leuven, Leuven, Belgium; Stanford Stroke Center, Palo Alto, CA, USA; Stanford Stroke Center, Palo Alto, CA, USA; Université Paris Cité, Institute of Psychiatrie and Neurosciences of Paris (IPNP), INSERM U1266, Team Turc, Paris, France; Neurology Department, CHU Montpellier, Montpellier, France; Radiology Department, Stanford University, Palo Alto, CA, USA; Neurology Department, CH Perpignan, Perpignan, France; Neurology Department, CHU Nimes, Nimes, France; Neurology Department, CH Béziers, Béziers, France; Neuroradiology Department, CHRU Gui de Chauliac, Montpellier, France; Stanford Stroke Center, Palo Alto, CA, USA; Stanford Stroke Center, Palo Alto, CA, USA

**Keywords:** Thrombolysis, thrombectomy, transfer, ischemic stroke

## Abstract

**Background:**

Patients with acute ischemic stroke with a large vessel occlusion (AIS-LVO) admitted to primary stroke centers (PSC) often require inter-hospital transfer to a comprehensive stroke center (CSC) for endovascular therapy (EVT). We aimed to determine the incidence of hemorrhagic transformation (HT) occurring during transfer, the factors associated with HT, and its relationship with 3-month outcome.

**Methods:**

We retrospectively analyzed data from two cohorts of AIS-LVO patients transferred from a PSC to a CSC for consideration of EVT. Patients were included if they had evidence of an anterior circulation AIS-LVO at the PSC and had a standard-of-care control brain imaging upon CSC arrival. HT was defined as any new hemorrhagic lesion within brain parenchyma visible on CSC admission imaging. Among HT patients, HT expansion was defined as an absolute volume increase of ⩾6 mL and a relative growth of ⩾33% between admission imaging and 24-h follow-up.

**Results:**

Overall, 566 patients were included, of whom 31 (5.5%) experienced HT during transfer. Inter-hospital HT was independently associated with inter-hospital arterial recanalization (adjusted odds ratio (aOR) = 6.95, 95%CI 2.94–16.39), higher pre-transfer NIHSS score (aOR = 1.08, 95%CI 1.02–1.14), and longer time from symptom onset to CSC arrival (aOR = 1.09, 95%CI 1.04–1.13). HT expansion between CSC arrival and 24 h occurred in 24% of HT cases. Inter-hospital HT was independently associated with modified Rankin scale ⩾3 at 3-month (aOR = 3.54, 95%CI 1.08–11.67, *p* = 0.038).

**Conclusion:**

HT during inter-hospital transfer for EVT is an uncommon event, yet is associated with a high rate of subsequent expansion and poor 3-month functional outcome. Treatments to reduce HT risk may be considered.

## Graphical Abstract

**Graphical abstract:**
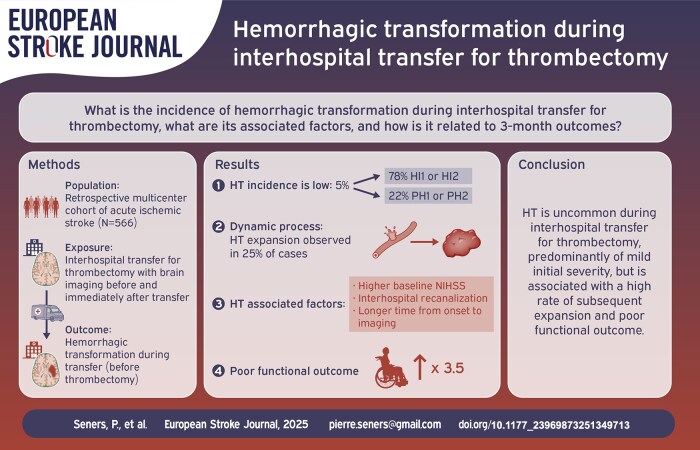

## Introduction

In patients with acute ischemic stroke due to large vessel occlusion (AIS-LVO), the benefits of endovascular therapy (EVT) are well established for up to 24 h after symptom onset.^1^ However, comprehensive stroke centers (CSCs) capable of performing EVT are scarce in most countries.^2^ As a result, up to two-thirds of AIS-LVO patients are initially assessed at community hospitals or primary stroke centers (PSCs) before being transferred to a CSC, a process that often results in treatment delays resulting in worse functional outcomes.^3,4^ Optimizing care for these patients is essential. A key first step is to enhance our understanding of the clinical and radiological changes occurring during transfer, as these may contribute to clinical outcomes.^4^

To date, inter-hospital infarct growth and the absence of recanalization during transfer have been consistently linked to poor 3-month outcomes, making them key therapeutic targets for future clinical trials.^4–9^ However, significant uncertainties remain regarding hemorrhagic transformation (HT) occurring during inter-hospital transfer. Its incidence is poorly documented,^10^ no studies have identified its clinical or imaging predictors, and its impact on 3-month functional outcomes remains unknown.^4^ This lack of data contrasts sharply with the extensive research on post-EVT HT, which occurs in 30%–50% of EVT-treated patients and has been well-characterized, with established associations with pre- or early post-treatment factors and a consistently negative effect on functional recovery.^11–16^

In this study, we aimed to determine the incidence of HT occurring during inter-hospital transfer for EVT, identify the clinical and imaging factors associated with inter-hospital HT, and assess its impact on 3-month outcomes. To achieve this, we analyzed a large multicenter cohort of AIS-LVO patients transferred for EVT, all of whom underwent brain imaging upon arrival at the CSC to evaluate HT.

## Methods

### Study design, data sources, and inclusion criteria

This retrospective study combined data from acute stroke patients consecutively admitted to two comprehensive stroke centers for consideration of EVT: Stanford Hospital, Palo Alto, USA (CRISP-2, an ongoing NIH funded prospective cohort study), and Montpellier Hospital, France (prospective registry). Details of these cohorts have been described in prior publications.^5,6,9,17^ Patients were included in the present analysis if they fulfilled the following criteria: (1) initial admission at a PSC where a standard-of-care MRI or CT showed an occlusion of the intracranial internal carotid artery (ICA), the first (M1) or second (M2) segment of the middle cerebral artery, (2) subsequent transfer to a CSC for EVT consideration, regardless of whether EVT was eventually attempted, and (3) standard-of-care follow-up brain imaging (MRI including T2* sequence, conventional non-contrast CT, or flat panel CT in the angio-suite) obtained upon admission in the CSC before any EVT procedure. Patients with an associated cervical carotid occlusion (i.e. tandem occlusion) or anterior cerebral artery occlusion were included. However, those with isolated cervical carotid occlusion or anterior cerebral artery occlusion were excluded. Patients lacking repeat CSC brain imaging, impairing HT assessment, were excluded. Inclusion dates were November 2019 to August 2024 for the US cohort and January 2015–January 2017 for the French cohort. These periods were selected because post-transfer brain imaging was required by protocol at both centers during these time frames. Patients were referred from 18 PSCs in the US cohort and from 5 PSCs in the French cohort. In all centers, acute stroke management followed international guidelines.

This study was reported according to the Strengthening the Reporting of Observational Studies in Epidemiology criteria for observational studies.^18^ The research was approved by the Stanford review board for the US Cohort and by the Rothschild Foundation Hospital review board for the French cohort. In the US Cohort, each participant provided informed consent. In the French cohort, the requirement for written informed consent was waived as this study only implied retrospective analysis of anonymized data collected as part of routine care. The data supporting the study findings are available upon reasonable request.

### Clinical data

The following variables were prospectively collected: age, sex, vascular risk factors, NIHSS score at the PSC, NIHSS score upon arrival at the CSC and at 24 h, intravenous thrombolysis (IVT) use, systolic blood pressure and serum glucose at the PSC, time from symptom onset (defined as last time seen well if unknown onset time) to PSC imaging and to CSC imaging, transfer duration (defined as the time between the PSC and CSC imaging, encompassing more than just transportation time), and pre-stroke and 3-month modified Rankin Scale (mRS) score. In the French cohort, NIHSS scores at the PSCs were assessed by on-site neurologists, whereas in the US cohort, assessments were performed by on-site or remote (telemedicine) neurologists or by emergency department physicians. The NIHSS score on CSC admission was evaluated by neurologists in both cohorts. Absolute change in NIHSS score during inter-hospital transfer was calculated as NIHSS_CSC_—NIHSS_PSC_; a negative value indicated clinical improvement and a positive value indicated clinical deterioration. Inter-hospital clinical improvement was defined as *a* ⩾4 NIHSS points decrease, and inter-hospital deterioration as *a* ⩾4 points increase. The 3-month mRS was assessed by a stroke neurologist or research nurse either during a face-to-face visit or by phone interview. Poor functional outcome at 3-month was defined as mRS 3–6.

### Radiological data

#### Primary stroke center

All included patients underwent either MRI or CT on admission at the PSC. Multimodal CT, including non-contrast CT, CT-angiography, and CT-perfusion was the routine first-line imaging technique for EVT candidates in all PSCs in the US cohort. MRI was the routine first-line imaging tool in all PSC in the French cohort, with a standardized protocol at each institution, which systematically included Diffusion-weighted imaging (DWI), T2*, and intracranial MR-angiography; perfusion imaging was rarely performed. One stroke neurologist with 14 years of clinical expertise in stroke imaging (P.S.) reviewed all imaging, blinded to clinical data. Intracranial occlusion site was assessed on MR- or CT-angiography, divided into ICA, M1, and M2, the M1 segment being defined as the first portion of the middle cerebral artery up to the main bifurcation. Infarct volume was measured on DWI or CT-perfusion. On DWI, infarct volume was manually outlined based on DWI signal intensity encompassing the entire area of bright DWI signal intensity. On CT-perfusion, infarct volume was automatically measured using RAPID software using the relative cerebral blood flow <30% of normal brain.

#### Comprehensive stroke center

During the study period, routine non-invasive post-transfer imaging was obtained as standard of care in both centers—before any EVT procedure. The routine first-line imaging technique was MRI in both cohorts, which included DWI, T2*, and intracranial MR-angiography. In rare instances patients went directly to the cathlab (i.e. bypassing the control non-invasive imaging) in case of short transfer time, at the discretion of the on-call physicians, and were therefore excluded unless flat panel CT was performed in the angio-suite before EVT. A follow-up brain imaging (CT or MRI) was obtained at 24 h in both cohorts. The change of intracranial arterial occlusion site during transfer was evaluated with head-to-head comparison of the PSC angiography (MR- or CT-angiography) and the initial CSC angiography (MR- or CT-angiography), and rated on the revised Arterial Occlusion Lesion score (rAOL, from 0, no recanalization to 3, complete recanalization).^19^ Inter-hospital recanalization was defined as an rAOL score of 2a–3, thereby including cases of both partial and complete recanalization.

#### Inter-hospital HT transformation assessment

Brain imaging performed upon CSC admission for all included patients was independently reviewed by two neurologists (P.S. and A.T.S.), with 14 and 9 years of experience in stroke imaging, respectively. Both readers were blinded to clinical data, and any discrepancies were resolved by consensus. HT was defined as any new hemorrhagic lesion within the brain parenchyma that was not visible on PSC imaging, excluding cerebral microbleeds. It was categorized according to the ECASS II (European Cooperative Acute Stroke Study) classification as hemorrhagic infarction (HI1 or HI2), parenchymal hemorrhage (PH1 or PH2), or remote parenchymal hemorrhage (rPH).^20^

To assess the evolution of HT over time, one reader (A.T.S) manually measured HT volume on CSC imaging (T2*-MRI or non-contrast CT) and 24-h follow-up imaging (T2*-MRI or non-contrast CT) using Horos (Horos Project, version 3.3.6), blinded to clinical outcomes. HT expansion was defined by an absolute HT volume increase of ⩾6 mL combined with a relative growth of ⩾33% between CSC imaging and 24-h follow-up, based on criteria from the primary intracerebral hemorrhage literature.^21^

### Statistical analysis

Continuous variables were described as mean ± standard deviation or median (interquartile range, IQR), as appropriate, and categorical variables as numbers and percentages. Interobserver agreement for inter-hospital HT classification was measured using weighted kappa statistics. Univariable relationships between inter-hospital HT and pre-transfer or immediate post-transfer characteristics were assessed using Student’s *t* test or the Mann-Whitney *U* test for continuous variables, and the Chi-square test or Fisher’s exact test for categorical variables, as appropriate. To adjust for potential confounders, multivariable binary logistic regression analysis was performed using a forward stepwise selection procedure with HT as the dependent variable. Covariates were entered into the multivariable regression model if they were significant at a threshold of *p* < 0.10 in the univariable analysis. Given the well-established higher sensitivity of T2*-MRI for detecting HT compared to CT, imaging modality was included as a forced covariate in the multivariable model. The relationship between inter-hospital HT and poor 3-month mRS was assessed using binary logistic regression, with poor 3-month mRS as the dependent variable. The model was adjusted for key confounders defined a priori based on a literature review, namely age, pre-stroke mRS, NIHSS score and core volume at PSC, onset-to-CSC admission time, IVT use, whether EVT was performed, and study center.^22^ In 59 patients with missing core volume due to the lack of CT-perfusion or MRI at the PSC, we imputed the baseline core volume using the value obtained from CT-perfusion or MRI performed upon arrival at the CSC. To explore the potential modifying effect of inter-hospital recanalization on the relationship between inter-hospital HT and poor 3-month outcome, we search for an HT*recanalization interaction. Results were reported as Odds Ratio (OR) and its 95%CI. All tests were two-tailed, and the threshold for statistical significance was set to *p* < 0.05. Statistical analyses were conducted using SPSS 28.0 (IBM, Armonk, NY).

## Results

### Study population

The combined US and French cohorts included 627 patients with anterior circulation LVO-related acute stroke who were transferred from a PSC for consideration of EVT. Among them, 61 (9.7%) patients were excluded due to the lack of HT assessment upon CSC admission (direct transfer to the angiosuite, *n* = 58; inadequate CSC admission imaging quality for HT assessment, *n* = 3), leaving 566 patients eligible for the study. The comparison between included and excluded patients is provided in Supplemental Table 1. Excluded patients had higher NIHSS score at PSC, larger PSC infarct core volume, and shorter transfer duration than included patients. Otherwise, both groups were comparable.

In the included population, median age was 72 years (IQR, 61–81) and 311 (55%) were male. At PSC admission, median NIHSS score was 15 (IQR, 10–20), onset-to-imaging time was 2.5 h (IQR, 1.3–7.3), and occlusion site was ICA in 111 (20%) patients, M1 in 340 (60%), and M2 in 115 (20%). IVT was administered before transfer in 274 (49%) patients, with alteplase 0.9 mg/kg in 248 and tenecteplase 0.25 mg/kg in 25. Median transfer time was 3.1 h (IQR, 2.5–3.8). Upon admission in the CSC, 141 (25%) patients had arterial recanalization (rAOL 2a–3). EVT was performed in 337 (60%) patients. A comparison of the main characteristics of the two participating sites is provided in Supplemental Table 2.

### Inter-hospital HT incidence and characteristics

On CSC admission, the repeat brain imaging to assess HT was T2*-MRI in 470 (83%) patients and non-contrast CT in 96 (17%) patients (conventional CT, *n* = 93; flat panel CT, *n* = 3). HT was observed in 31 patients (5.5%, 95%CI 3.6–7.4). There was a trend toward a lower HT rate when assessed on CT than on T2*-MRI (2/96 = 2.1% vs 29/470 = 6.2%, respectively, *p* = 0.11). There was substantial agreement between the two readers for HT classification (weighted kappa, 0.79 (95%CI, 0.67–0.91)). HT subtypes were HI1 in 8/31 (26%) patients, HI2 in 16/31 (52%) patients, PH1 in 3/31 (10%), PH2 in 2/31 (6%), and remote PH in 2/31 (6%). The two HTs detected on CT were HI1.

### HT evolution between CSC admission and 24-h follow-up

**Figure 1.**
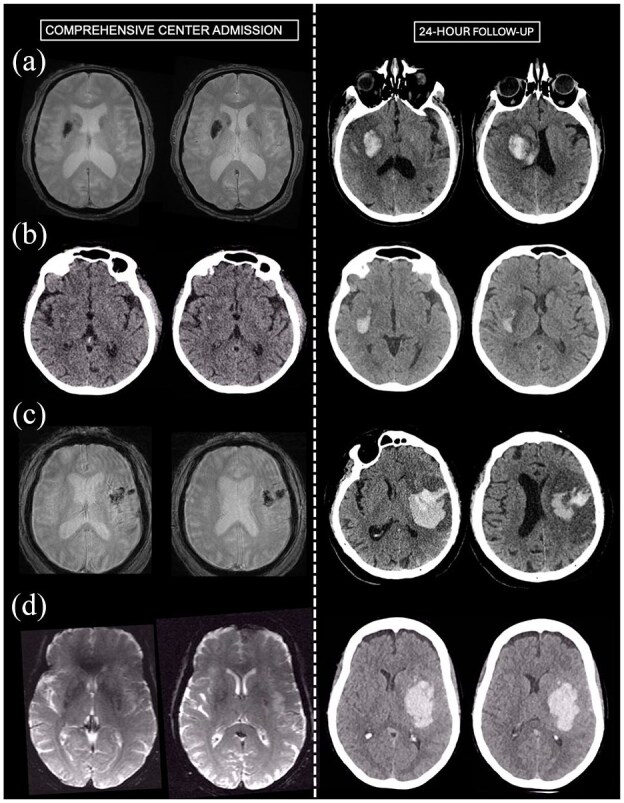
Representative cases with inter-hospital hemorrhagic transformation and subsequent expansion. (a) Patient admitted in the PSC with NIHSS of 22 and M1 occlusion. IVT was administered. Brain MRI on CSC admission 3.5 h later (NIHSS 20) shows an M1 recanalization (distal middle cerebral artery branch occlusion, rAOL 2b recanalization) and a deep HI-2 HT on T2*. EVT was not performed. 24-h follow-up CT shows HT expansion (from 6 to 15 cc). (b) Patient admitted in the PSC with NIHSS of 12 and M1 occlusion. IVT was administered. CT on CSC admission 2.5 h later (NIHSS 8) shows an M1 recanalization (distal middle cerebral artery branch occlusion, rAOL 2b recanalization) and a deep HI-1 HT. EVT was not performed. 24-h follow-up CT shows HT expansion (from <1 to 8 cc). (c) Patient admitted in the PSC with NIHSS of 16 and proximal M2 occlusion. IVT was administered. Brain MRI on CSC admission 3.5 h later (NIHSS 17) shows a stable M2 occlusion and a PH-1 HT on T2*. EVT was not performed. 24-h follow-up CT shows HT expansion (from 12 to 57 cc). (d) Patient admitted in the PSC with NIHSS of 17 and M1 occlusion. IVT was not administered. Brain MRI on CSC admission 3.7 h later (NIHSS 13) shows a slight M1 thrombus migration (rAOL 1) and a deep HI-2 HT on T2*. EVT was performed, with modified Thrombolysis In Cerebral Infarction score 2b achieved after one pass. 24-h follow-up CT shows HT expansion (from 1 to 77 cc). CSC: comprehensive stroke center; EVT: endovascular therapy; HI: hemorrhagic infarction; HT: hemorrhagic transformation; IVT: intravenous thrombolysis; PH: parenchymal hematoma; PSC: primary stroke center; M1 and M2: first, and second segment of the middle cerebral artery occlusion; rAOL: revised Arterial Occlusive Lesion scale.

Follow-up imaging at 24-h was available in 29/31 HT cases. Imaging type for HT expansion assessment was CT-to-CT in 2 patients, MRI-to-MRI in 10 patients and MRI-to-CT in 17 patients. HT expansion between CSC admission and 24 h follow-up occurred in 7/29 (24%) patients. Among these seven patients, the initial HT at CSC admission was classified as HI1 in one case, HI2 in four cases, and PH1 in two cases. Among HT patients, EVT was not associated with subsequent HT expansion (27% (3/11) in EVT patients vs 22% (4/18) in non-EVT patients). Similarly, IVT was not associated with HT expansion (57% (4/15) in IVT patients vs 43% (3/14) in non-IVT patients). Representative HT cases with HT expansion are shown in Figure 1.

### Factors associated with inter-hospital HT

As compared to patients without HT, those with inter-hospital HT had higher NIHSS score at PSC, longer transfer duration, longer time from symptom onset to CSC admission, and higher inter-hospital recanalization rates (Table 1). IVT rate was similar across the two groups (no IVT: 14/292 = 4.8%; IVT: 17/274 = 6.2%, *p* = 0.46). The rate of PH 1 or 2 was numerically higher in the IVT group, but the difference was not significant (no IVT: 2/292 = 0.7%; IVT: 5/274 = 1.8%, *p* = 0.27). EVT rate was lower in patients with inter-hospital HT as compared to those without HT (36% vs 61%, *p* < 0.01). Among the 20 patients with inter-hospital HT, EVT was not performed due to recanalization in 12 cases (60%), a large ischemic core in 2 cases (10%), HT alone in 3 cases (15%—all with PH1 or PH2), and a combination of large core and HT in the remaining 3 cases (15%).

**Table 1.** Univariable comparison of pre-transfer and immediate post-transfer characteristics according to the hemorrhagic transformation status.

Variables	HT (*N* = 31)	No HT (*N* = 535)	*p*-value	Missing data
Age	72 (61–82)	72 (61–81)	0.88	0
Male	18 (58)	293 (55)	0.72	0
Hypertension	19 (61)	357 (67)	0.49	4
Diabetes	7 (23)	119 (22)	0.97	2
Dyslipidemia	12 (39)	213 (40)	0.87	5
Pre-stroke antiplatelets	13 (42)	162 (31)	0.21	13
Pre-stroke anticoagulant	2 (7)	88 (17)	0.13	11
CSC site			0.40	0
Stanford	22 (6)	340 (94)		
Montpellier	9 (4)	195 (96)		
Clinical characteristics (PSC)				
NIHSS score	17 (13–22)	14 (9–20)	**0.01**	11
Glucose, mg/dL	127 (112–143)	119 (104–144)	0.39	4
Systolic blood pressure, mmHg	148 (133–164)	147 (128–164)	0.90	4
Imaging characteristics (PSC)				
Onset-to-imaging, h	3.1 (1.6–8.2)	2.4 (1.3–7.0)	0.26	2
Occlusion site			0.12	0
ICA	6 (19)	105 (20)		
M1	23 (74)	317 (59)		
M2	2 (7)	113 (21)		
Associated cervical ICA occlusion	5 (16)	101 (19)	0.70	0
Infarct core volume, mL (*n* = 507)^a^	11 (0–32)	9 (0–22)	0.76	60
IV-thrombolysis use	17 (54)	257 (48)	0.46	0
CSC arrival				
Transfer time, h	3.7 (3.3–4.4)	3.0 (2.5–3.8)	**<0.01**	4
Onset-to-CSC arrival time, h	6.8 (5.5–12.4)	6.1 (4.5–10.9)	**0.05**	6
Recanalization during transfer^b^	16 (53)	125 (23)	**<0.01**	1
Underwent thrombectomy	11 (36)	326 (61)	**<0.01**	0

Categorical variables are expressed as numbers (%) and continuous variables as median (interquartile range). Bold *p*-values indicate *p*-values < 0.05.

CSC: comprehensive stroke center; HT: hemorrhagic transformation; ICA: intracranial internal carotid artery; IV: intravenous; M1 and M2: first and second segment of the middle cerebral artery; PSC: primary stroke center.

^a^Infarct volume was measured on diffusion-weighted imaging (manual delineation) or CT-perfusion (relative cerebral blood flow < 30%).

^b^Inter-hospital recanalization was defined as a revised AOL score 2a, 2b, or 3.

In multivariable analysis, inter-hospital HT was independently associated with higher NIHSS score at PSC, longer onset-to-CSC imaging time and inter-hospital recanalization (Table 2). There was a non-significant trend toward an association with imaging modality.

**Table 2.** Logistic regression model showing variables independently associated with inter-hospital hemorrhagic transformation.

	Unadjusted OR (95%CI)	Adjusted OR (95%CI)	*p*-value
Inter-hospital recanalization (rAOL 2a–3)	3.75 (1.78–7.89)	6.95 (2.94–16.39)	<0.001
Onset-to-CSC arrival time	1.05 (1.02–1.09)	1.09 (1.04–1.13)	<0.001
NIHSS score before transfer	1.07 (1.01–1.14)	1.08 (1.02–1.14)	0.013
Imaging modality to detect HT is MRI	3.09 (0.73–13.2)	3.95 (0.88–17.75)	0.07

CSC: comprehensive stroke center; HT: hemorrhagic transformation; OR: odds ratio; rAOL: revised arterial occlusive lesion score.

### Relationship between inter-hospital HT and clinical outcomes

**Figure 2.**
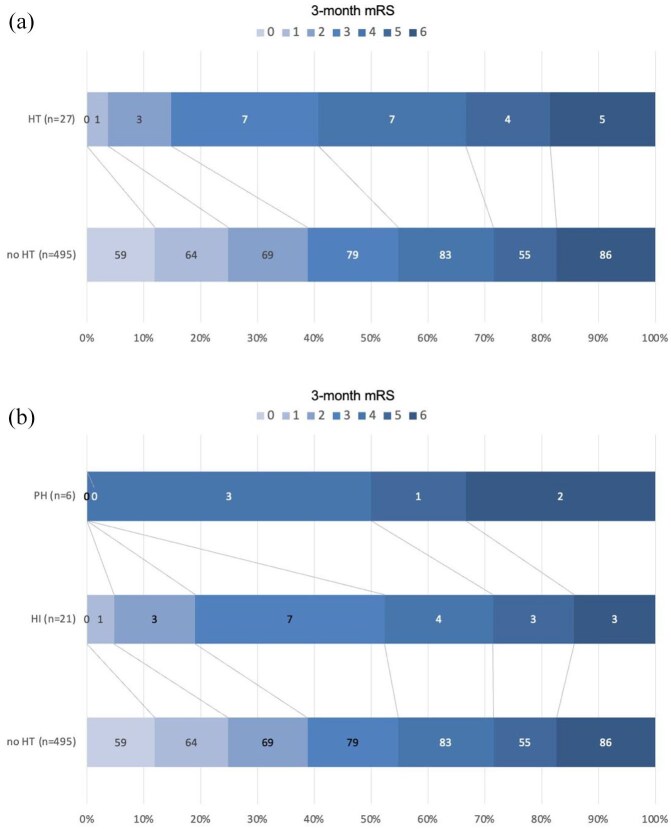
Relationship between inter-hospital hemorrhagic transformation and 3-month functional outcome. (a) Three-month mRS according to inter-hospital HT status. Inter-hospital HT was associated with lower rate of poor functional outcome (mRS >2, adjusted odds ratio (OR) = 3.54, 95%CI 1.08–11.67, *p* = 0.038), adjusted for age, pre-stroke mRS, NIHSS score and core volume at PSC, onset-to-CSC admission time, IVT use, whether EVT was attempted, and study center. (b) Three-month mRS according to inter-hospital HT status, stratified according to HT severity. The 3-month mRS was missing in 44 of 566 (9%) patients (4/31 (13%) with HT and 40/535 (8%) without). HI indicates hemorrhagic infarction; HT, hemorrhagic transformation; mRS, modified Rankin Scale score; PH, parenchymal hematoma.

The association between inter-hospital HT and clinical outcomes are shown in Table 3 and Figure 2. Upon CSC admission, inter-hospital HT was associated with higher rates of inter-hospital clinical improvement, but at the 24 h time-point, there was a trend toward higher NIHSS score in patients with inter-hospital HT. Inter-hospital clinical improvement was primarily observed in patients with mild HT upon CSC arrival (HI1 or 2:14/23 = 61%; PH: 2/7 (29%); no HT: 130/515 (24%), *p* < 0.001). Clinical deterioration during transfer was uncommon among patients with HT and did not differ significantly from those without HT (HT: 4/31 = 13% vs no HT: 78/515 = 15%, *p* = 0.79).

**Table 3.** Comparison of clinical outcomes according to the hemorrhagic transformation status.

	HT (*N* = 31)	No HT (*N* = 535)	*p*-value	Missing data
Comprehensive stroke center arrival				
NIHSS score	14 (10–20)	13 (8–19)	0.37	10
NIHSS change during transfer^a^	−4 (−8, 0)	0 (−4, 2)	**0.02**	21
Inter-hospital clinical deterioration^a^	4 (13)	78 (15)	0.79	21
Inter-hospital clinical improvement^a^	16 (53)	130 (25)	**<0.01**	21
24-h				
NIHSS score	11 (6–18)	9 (3–17)	0.07	6
NIHSS change from CSC admission^b^	-1 (−5, 1)	−3 (−6, 0)	0.27	15
3-month				
mRS score	4 (3–5)	3 (2–5)	**0.05**	44
mRS 3–6	23 (85)	303 (61)	**0.01**	44

Categorical variables are expressed as numbers (%) and continuous variables as median (interquartile range). Bold *p*-values indicate *p*-values < 0.05.

HT: hemorrhagic transformation; mRS: modified Rankin score.

^a^Absolute change in NIHSS score during inter-hospital transfer was calculated as NIHSS_CSC_—NIHSS_PSC_; a negative value indicated clinical improvement and a positive value indicated clinical deterioration. Inter-hospital clinical improvement was defined as *a* ⩾4 NIHSS points decrease, and inter-hospital deterioration as *a* ⩾4 points increase.

^b^Absolute change in NIHSS score between CSC admission and 24 h was calculated as NIHSS_24h_—NIHSS_CSC_; a negative value indicated clinical improvement and a positive value indicated clinical deterioration.

Poor functional outcome (defined as mRS equal or greater than 3) occurred in 326/522 (63%) patients with available 3-month mRS, with higher rates among patients with versus without inter-hospital HT (23/27 = 83% vs 303/495 = 61%, *p* = 0.01). The difference remained significant following adjustment for age, pre-stroke mRS, NIHSS score and core volume at PSC, onset-to-CSC admission time, IVT use, whether EVT was attempted, and study center (adjusted OR = 3.54, 95%CI 1.08–11.67, *p* = 0.038). No inter-hospital HT*inter-hospital recanalization was observed (P for interaction = 0.81), indicating that the association between inter-hospital HT and poor 3-month outcome was not influenced by recanalization status during transfer.

As compared to patients with HI1 or HI2, patients with PH had numerically poorer outcome (Figure 2(b)). All the patients with HT expansion post transfer had poor 3-month functional outcomes. Subgroup analyses focusing on patients with PH or those with HT expansion could not be performed due to the low number of events. In an alternative model excluding the seven patients with PH, no association was observed between HI1 or HI2 and poor outcome (OR = 2.30, 95%CI 0.67–7.89, *p* = 0.19, adjusted for the same confounders).

## Discussion

Based on a large cohort of patients with anterior circulation AIS-LVO transferred from a PSC to a CSC for EVT evaluation, this study highlights four key findings. First, the incidence of inter-hospital HT is low and predominantly mild in severity, with HI1 or HI2 accounting for 75% of cases. Second, HT is often a dynamic process, with substantial expansion observed at 24-h follow-up in 24% of cases. Third, inter-hospital HT is independently associated with a higher NIHSS score at the PSC, longer onset-to-CSC imaging time, and inter-hospital recanalization. Last, inter-hospital HT is associated with a fourfold increase in the odds of poor functional outcome at 3 months.

The rate of inter-hospital HT in our cohort was low (5.5%) but notably higher than the only previous study reporting similar findings (12/836, 1.4%).^10^ This discrepancy may stem from differences in imaging modalities used to assess HT, though the previous study did not specify its imaging method. In our cohort, T2*-MRI, which is known to be more sensitive than non-contrast CT for HT detection,^23^ was used in 83% of cases. Consistently, HT rates were lower when assessed by CT than by T2*-MRI (2.1% vs 6.2%, respectively). Our study is the first to describe the radiological characteristics of HT. We found that inter-hospital HT is initially mild in most cases, with 75% classified as HI1 or HI2. However, we also found that substantial HT expansion occurred in 24% of cases between CSC admission and the 24-h follow-up, paralleling hematoma expansion patterns in acute primary intracerebral hemorrhage.^21,24^ The findings suggest that the HT observed upon arrival at the CSC is at an early stage of a dynamic process and highlights that a small hemorrhage may be misleadingly reassuring. A limitation is the use of different imaging modalities for HT volume measurement in 58% of cases, primarily MRI at CSC admission followed by CT at 24 h. In primary intracerebral hemorrhage, hematoma volume tends to appear larger on MRI than on CT, though the absolute volumetric difference has been shown to be small.^25^ As a result, HT expansion may have been underestimated in our cohort. In our study, inter-hospital HT was associated with lower EVT rates (36% vs 61%). This is partly due to higher inter-hospital recanalization rates among patients with HT obviating the need for EVT and in some cases due to concerns regarding the safety of EVT in the setting of HT. Although EVT was not associated with subsequent hematoma expansion, the small sample size limits definitive conclusions. The safety and efficacy of EVT in patients with mild HT therefore remains uncertain and requires further investigation.

Inter-hospital HT was independently associated with greater baseline clinical severity and a longer time from symptom onset to CSC imaging, aligning with the well-established link between these factors and post-IVT or post-EVT HT assessed at 24 h.^15,26,27^ Additionally, we found that recanalization occurring during transfer was associated with a sevenfold increased risk of inter-hospital HT. This finding is consistent with previous studies in IVT-treated populations, which reported an association between HT —mostly of mild radiological severity at 24 hours— and recanalization or brain reperfusion.^27–31^ These data suggest that arterial recanalization may be a double-edged sword: while it is beneficial in most cases by salvaging ischemic brain tissue, it may also promote HT in some patients. Notably, inter-hospital bleeding was associated with clinical improvement—not clinical deterioration. This apparent paradox is likely explained by the higher rate of recanalization in HT patients. When inter-hospital recanalization occurs, the clinical benefits of penumbral tissue salvage may outweigh any negative impact of HT on the NIHSS. The absence of an association between inter-hospital HT and clinical deterioration is consistent with previous reports, where HT-related worsening was nearly absent.^10,17,32^ Moreover, one study found that IVT-related HI1-HI2 at 24 h was associated with early recanalization and short-term clinical improvement.^28,33^ Importantly, IVT use was not linked to inter-hospital HT in our cohort, consistent with observational studies showing comparable 24 h HT rates between IVT-treated and non-IVT-treated AIS-LVO patients transferred from a PSC for EVT.^34^ Of note, we observed a numerically higher rate of PH1 and PH2 in the IVT group; however, this difference was not statistically significant, possibly due to limited statistical power.

Despite its association with clinical improvement upon CSC arrival, inter-hospital HT was independently associated with worse functional outcomes at 3 months, even after adjustment for key confounders. However, in our study, milder forms of HT (HI1 or HI2) were not significantly associated with poor outcomes, which may be explained by limited statistical power. Nonetheless, emerging evidence suggests that even mild HT—when detected 24 h after IVT or EVT and in the absence of immediate clinical deterioration—can negatively impact long-term functional recovery.^12,13,35–37^ This finding may be particularly relevant when mild HT is identified early after inter-hospital transfer for EVT —as done in our study—, potentially reflecting an early stage of HT. This is underscored by the substantial rate of subsequent HT expansion observed in our study (24%). Further large-scale studies are needed to clarify the potential association between inter-hospital HI and poor 3-month outcomes. Therapeutic strategies aimed at preventing its occurrence or limiting its progression—such as interventions targeting neuroinflammation, thromboinflammation, or blood-brain barrier damage—merit further investigation.^38,39^

The necessity of repeating brain imaging upon CSC admission in transferred AIS-LVO patients remains a subject of debate. Imaging findings that may justify aborting EVT in transfer patients include arterial recanalization and HT, both of which are associated with inter-hospital clinical improvement. Therefore, repeating imaging in patients with clinical improvement during transfer appears warranted to avoid unnecessary invasive procedures. To minimize delays in reperfusion caused by repeat conventional imaging, cone-beam non-contrast CT and CT perfusion in the angiosuite have emerged as a promising alternative.^40^ The ANGIOCAT trial (Evaluation of Direct Transfer to Angiography Suite vs Computed Tomography Suite in Endovascular Treatment: Randomized Clinical Trial) assessed the safety and efficacy of direct transfer to the angiosuite —bypassing conventional imaging and using on-table flat-panel non-contrast CT to exclude HT—.^41^ Notably, 75% of enrolled patients were inter-hospital transfers for EVT, all with a persistent NIHSS score > 10 upon CSC arrival. The study demonstrated that, in this situation, direct transfer to the angiosuite significantly reduced door-to-reperfusion time and improved 3-month functional outcomes without compromising safety.

This study has several limitations. First, 10% of patients were excluded due to the lack of inter-hospital HT assessment, which may have introduced bias into our sample. Second, the imaging modality used to assess HT was not standardized, with 83% of patients undergoing MRI and 17% CT. Given the higher sensitivity of MRI compared to CT for detecting HT, this heterogeneity may have introduced bias into our findings. In particular, for patients who had a CT at PSC followed by an MRI upon admission to the CSC, some HTs identified at the CSC might have already been present but went undetected on the initial CT due to its poorer sensitivity. Third, as previously discussed, HT volumes were compared across different imaging modalities in two-thirds of cases, which may have limited the accuracy of assessing HT expansion over time. Fourth, data on blood pressure variability or management during transfer, which could influence HT occurrence, were not collected. Also, the relationship between inter-hospital HT and potentially relevant imaging biomarkers—such as markers of small vessel disease or the severity of hypoperfusion—could not be assessed due to the lack of consistent imaging data across the entire cohort. Fifth, the median transfer time (defined as the time between the PSC and CSC imaging, encompassing more than just transportation time) in our study was relatively long, as reported in other settings.^8,42^ Results may differ in regions with shorter transfer times. Sixth, some differences were observed between the French and US cohorts. However, the inter-hospital HT rate was similar across the two cohorts, and the outcome analysis was adjusted for study center. Finally, due to the observational nature of the study, a causal relationship between inter-hospital HT and poor functional outcome cannot be established.

## Conclusion

This study demonstrates that HT is uncommon (5%) in AIS-LVO patients transferred from a PSC to a CSC for evaluation of EVT, and predominantly of mild initial severity (HI1 or HI2 in 75% of cases). However, inter-hospital HT is associated with a high rate of subsequent expansion and poor functional outcomes at 3 months, particularly among patients who develop parenchymal hematomas. Inter-hospital HT is linked to higher baseline NIHSS scores, longer time from symptom onset, and higher rates of inter-hospital recanalization. There is a role for the development of inter-hospital strategies and treatments that reduce the risk of HT.

## Supplementary Material

sj-docx-1-eso_23969873251349713

## Data Availability

The data supporting the study findings are available from the corresponding author upon reasonable request.
