# Supplementary material for: Hemorrhagic transformation during inter-hospital transfer for thrombectomy: Incidence, associated factors, and relationship with outcome
Source: Eur Stroke J. 2026 Jan 1;11(1):23969873251349713. doi: 10.1093/esj/23969873251349713 (PMC12866271; doi:10.1093/esj/23969873251349713)
Supplement: sj-docx-1-eso_23969873251349713 [file sj-docx-1-eso_23969873251349713.docx]

**SUPPLEMENTAL MATERIALS**

**Supplemental Table 1 – Comparison included and excluded (lack of hemorrhagic transformation assessment) patients**

|  | **Included**  **N=566** | **Excluded**  **N=61** | ***P*-value** |
| --- | --- | --- | --- |
| Age | 72 (61-81) | 74 (66-81) | 0.27 |
| Male | 311 (55) | 32 (53) | 0.71 |
| Hypertension | 376 (67) | 45 (75) | 0.20 |
| Diabetes | 126 (22) | 10 (17) | 0.31 |
| Dyslipidemia | 225 (40) | 18 (30) | 0.11 |
| Pre-stroke antiplatelets | 175 (32) | 18 (30) | 0.73 |
| Pre-stroke anticoagulant | 90 (16) | 12 (20) | 0.49 |
| **Clinical characteristics (PSC)** |  |  |  |
| NIHSS score | 15 (10-20) | 17 (12-21) | **0.01** |
| Glucose, mg/dL | 120 (104-144) | 120 (106-144) | 0.72 |
| Systolic blood pressure, mmHg | 147 (128-164) | 149 (128-168) | 0.45 |
| **Imaging characteristics** **(PSC)** |  |  |  |
| Onset-to-imaging, hrs | 2.5 (1.3-7.3) | 2.2 (1.6-7.5) | 0.91 |
| Occlusion site |  |  | 0.64 |
| ICA | 111 (20) | 15 (25) |  |
| M1 | 340 (60) | 35 (57) |  |
| M2 | 115 (20) | 11 (18) |  |
| Associated cervical ICA occlusion | 106 (19) | 12 (20) | 0.86 |
| Infarct core volume, mL^a^ | 9 (0-22) | 16 (5-44) | **<0.01** |
| IV-thrombolysis use | 274 (49) | 28 (46) | 0.70 |
| **CSC arrival** |  |  |  |
| Transfer time, hours | 3.1 (2.5-3.8) | 2.6 (2.3-3.2) | **<0.01** |
| Onset-to-CSC arrival time, hrs | 6.2 (4.5-11.2) | 5.0 (4.5-8.3) | 0.08 |
| Recanalization during transfer^b^ | 141 (25) | 16 (27) | 0.72 |
| NIHSS change during transfer^c^ | 0 (-4, 2) | 0 (-2, 2) | 0.16 |
| Inter-hospital deterioration^c^ | 82 (15) | 13 (22) | 0.14 |
| Inter-hospital improvement^c^ | 146 (27) | 11 (19) | 0.20 |

Categorical variables are expressed as numbers (%) and continuous variables as median (interquartile range).

a: Infarct volume was measured on diffusion-weighted imaging (manual delineation) or CT-perfusion (relative cerebral blood flow <30%). Available in 507 included patients and 59 excluded patients.

b: Inter-hospital recanalization was defined as a revised AOL score 2a, 2b or 3.

c: Absolute change in NIHSS score during inter-hospital transfer was calculated as NIHSS_CSC_ – NIHSS_PSC_; a negative value indicated clinical improvement and a positive value indicated clinical deterioration. Inter-hospital clinical improvement was defined as a ≥4 NIHSS points decrease, and inter-hospital deterioration as a ≥4 points increase.

CSC indicates comprehensive stroke center; HT, hemorrhagic transformation; ICA, intracranial internal carotid artery; IV, intravenous; M1 and M2, first and second segment of the middle cerebral artery; PSC, primary stroke center.

**Supplemental Table 2.** Comparison of main characteristics in the two participating sites

|  | **Montpellier, France**  **(n=204)** | **Stanford, USA**  **(n=362)** | **P-value** |
| --- | --- | --- | --- |
| Age | 72 (60-80) | 72 (62-81) | 0.54 |
| Male | 110 (54) | 201 (56) | 0.71 |
| NIHSS score (referring hospital) | 15 (10-20) | 14 (9-20) | 0.36 |
| Last seen well-to-imaging time, hours | 2.3 (1.6-4.2) | 2.9 (1.1-8.7) | 0.32 |
| Occlusion site |  |  | <0.01 |
| ICA | 46 (23) | 65 (18) |  |
| M1 | 134 (65) | 206 (57) |  |
| M2 | 24 (12) | 91 (25) |  |
| Associated cervical ICA occlusion | 38 (19) | 68 (19) | 0.96 |
| IV-thrombolysis use | 134 (66) | 140 (39) | <0.01 |
| Transfer time, hours | 2.9 (2.5-3.4) | 3.3 (2.5-4.2) | <0.01 |
| HT on CSC admission | 9 (4) | 22 (6) | 0.40 |
| Inter-hospital recanalization (rAOL 2a-3) | 57 (28) | 84 (23) | 0.20 |

Categorical variables are expressed as numbers (%) and continuous variables as median (interquartile range).
